# Evolution of Knowledge, Awareness, and Practices regarding Zika Virus from 2016 to 2017

**DOI:** 10.1155/2017/6350602

**Published:** 2017-11-20

**Authors:** Quinton Katler, Prachi Godiwala, Charles Macri, Beth Pineles, Gary Simon, Aileen Chang, Homa Ahmadzia

**Affiliations:** ^1^Department of Obstetrics and Gynecology, The George Washington University Hospital, Washington, DC, USA; ^2^Department of Obstetrics, Gynecology and Reproductive Sciences, University of Maryland Medical Center, Baltimore, MD, USA; ^3^Department of Internal Medicine, The George Washington University Hospital, Washington, DC, USA

## Abstract

**Objective:**

Our team created a knowledge, attitudes, and practice (KAP) survey in order to assess changes over time in healthcare provider and community member awareness of Zika virus symptoms, transmission, treatment, and current and future concerns.

**Study Design:**

The cross-sectional survey was issued at an academic medical center in Washington, DC, and via an online link to healthcare providers and community members between June and August 2016. Survey distribution was then repeated the following year, from March to April 2017. Outcomes were compared by survey year and healthcare provider versus community member status using SAS Program Version 9.4.

**Results:**

Significant differences in knowledge, attitudes, and practices existed between 2016 and 2017 survey time points. By 2017, more respondents had knowledge of various Zika virus infection characteristics; however healthcare provider knowledge also waned in certain areas. Attitudes towards Zika virus infection displayed an overall decreased concern by 2017. Practice trends by 2017 demonstrated fewer travel restrictions to Zika-endemic areas and increased mosquito protective measures within the US.

**Conclusions:**

Our results provide novel insight into the transformation of knowledge, attitudes, and practice of community members and healthcare providers regarding Zika virus since its declaration as a public health emergency of international concern in 2016.

## 1. Introduction

Zika virus infection was declared a public health emergency in 2016. Zika virus infections have been present in Africa and Asia since the 1940s and have since spread dramatically to mainly in the Americas and western Pacific [1]. Zika virus was first identified in a rhesus monkey in Uganda in 1947, during which time the virus vector was discovered as the* Aedes africanus* mosquito [2–4]. Since the initial detection of Zika virus, epidemiologists have mapped its global migration through the remainder of the 20th century [5]. In October of 2015, Brazil reported “an unusual increase” in the number of cases of microcephaly among newborns. After increased investigative efforts, Brazil reported detection of Zika virus in amniotic fluid samples from pregnant women with ultrasound-confirmed microcephaly, culminating in the issuance of a national public health emergency [6]. Zika virus reached the United States (US) in mid-2016 when the first local-transmission was documented in Miami on July 29, 2016 [7]. As of July 2017, there had been over 143 symptomatic Zika virus disease cases reported within the US and over 513 cases within US Territories [8].

Since the first documented cases in the western hemisphere, the scientific community has made substantial progress in terms of understanding Zika virus transmission and outcomes. It is known that an infected mother can pass the Zika virus vertically to her fetus during pregnancy as well as during the delivery of her infant [9]. The Zika virus can also be passed through sexual intercourse, even if the infected person is asymptomatic [9]. There are reports of Zika virus infections acquired through laboratory exposure [9]. Other modes of transmission are being investigated, such as blood transfusion and healthcare occupational exposure. The most common symptoms of a Zika infection include fever, rash, headache, joint pain, conjunctivitis, and muscle pain. Zika virus has been associated with specific patterns of neurologic and anatomic abnormalities in newborns, including microcephaly (congenital small head size leading to brain abnormalities), decreased brain tissue volume, and ocular damage [9]. Zika infection is diagnosed by screening of symptoms and recent travel history followed by testing of blood or urine. While there is no commercially available vaccine or targeted treatment for Zika virus, symptoms are treated by rest, hydration, and fever-reducing medication [9].

Despite substantial progress in understanding Zika virus pathophysiology among the scientific community, to our knowledge there is no study or data regarding the evolution in knowledge, awareness, and practice among community members and healthcare providers with regard to Zika virus during this time period. Given this void, the aim of our study was to analyze the change in knowledge, awareness, and prevention/practice regarding Zika infection during a critical time in the development of understanding of the virus.

## 2. Study Design

A Zika questionnaire was composed by a multidisciplinary team of experts consisting of maternal-fetal-medicine physicians, public health specialists, general medicine providers, and obstetrician-gynecologist generalist physicians. This cross-sectional survey was distributed in two platforms. First, patients and other community members visiting the outpatient obstetrics and gynecology clinics at a mid-size academic center in Washington, DC, were approached by medical student study volunteers to take the survey prior to their scheduled appointments over a 6-week period from late June to early August 2016. This time period was chosen because, during this interval, information about Zika virus and practices to safely prevent transmission were at the forefront of media attention due to many uncertainties surrounding the virus, and it is a common time for our study participants to be traveling to areas prevalent with Zika virus. The survey was loaded onto handheld tablet computers via the secure REDCap platform and volunteers approached respondents at random during normal clinic hours; the intervals of office survey administration were dependent on the availability of volunteers. Second, the survey was emailed to community members and healthcare providers using REDCap to collect the data. The REDCap survey link was emailed to various listservs within the academic medical community, including OB/GYN resident and faculty listservs, medical student listservs, and the Rodham Institute. The survey was also emailed to nonmedical personnel via social media networks in order to obtain a random sampling of community members. The survey distribution was then repeated in the same fashion over 6 weeks nearly one year later, from early March to late April 2017. This time period was chosen because the authors felt that sufficient time had passed since the first survey administration to gauge a change in knowledge, attitudes, and practices; Zika virus garnered much less media attention around this time due to increased availability of information regarding the virus.

Healthcare providers were defined as anyone involved in the medical field and were stratified as either a physician (primary care, OB/GYN, or other specialty physicians), or nonphysician, which could include but was not limited to medical students, NPs, PAs, genetic counselors, and other types of providers. Information about the type of nonphysician healthcare providers was not collected. Community members were defined as anyone receiving care at the clinic where the survey was administered or anyone whose occupation was not in the healthcare field. The survey sample size included 174 respondents (63% community members; 37% healthcare providers) during the first round and 277 respondents (65% community members; 35% healthcare providers) during the second round. No face-to-face interviews were conducted. At the end of the survey the respondent was provided with a link to the most current CDC Guidelines on Zika virus.

Data was stored in the George Washington University REDCap database. Outcomes were compared by year of survey administration and healthcare provider versus community member status. Data analysis was performed using SAS Program Version 9.4. The median and interquartile range was computed for continuous variables. Frequencies were computed for categorical variables. Chi-square tests were used to compare frequencies of categorical variables across groups. *p* values < 0.05 were considered statistically significant.

The study was approved by the Office of Human Research-Institutional Review Board at The George Washington University (Protocol #031653, approved April 20, 2016). Respondent identity was anonymous to protect confidentiality.

## 3. Results

Of the approximately 800 individuals approached to take the survey either in the clinic or via email link in each round, approximately half were healthcare providers and half were community members, and 451 potential participants responded. Approximately 75 of these were community members approached in the waiting room of the clinic. Of the 451 total survey respondents, there were more respondents in the second round of surveys (277) compared to the first (174). Fifty-seven respondents (33%) in the first round of surveys were healthcare providers and 60% of these were physicians, whereas 89 (32%) were healthcare providers in the second round but only 37% of these were physicians. The population surveyed was majority Caucasian females with private medical insurance (Table 1).

Significant differences in knowledge existed between 2016 and 2017 survey time points (Table 2). More community members were aware of Zika virus in the second round of surveys (99% in 2017 versus 94% in 2016, *p* = 0.02). More respondents were able to correctly identify Zika infection symptoms in 2017, and this remained true for both community members (64% versus 46%, *p* = 0.019) as well as healthcare providers (79% versus 74%, *p* = 0.19). When asked if anyone had been infected with Zika virus within the US at the time of survey collection, more community members were aware in 2017 compared to 2016 (91% versus 65%,* p* ≤ 0.0001), and a similar trend was noted for healthcare providers (84% versus 61%, *p* = 0.002). Knowledge with regard to a link between Zika infection during pregnancy and microcephaly increased among community respondents (95% in 2017 versus 90% in 2016, *p* = 0.09), and this knowledge remained basically unchanged among healthcare providers (100% versus 98%, *p* = 0.25). Knowledge about safety of mosquito-bite prevention strategies such as DEET and permethrin during pregnancy actually decreased over time, including for both healthcare providers and community members with a lower proportion of responses indicating knowledge of DEET and permethrin safety in pregnancy in 2017. However, a substantial number of respondents answered “I do not know” to this question in both years. Knowledge regarding effective protective measures against mosquitos changed among community members over time: in 2017, 93% (versus 82% in 2016) would remove standing water (*p* = 0.015), 96% (versus 78%) would sleep under a mosquito net (*p* ≤ 0.0001), and 96% (versus 85%) would use screens on windows and doors (*p* = 0.0049); healthcare provider knowledge essentially remained the same between the survey time points.

Community member knowledge appears to increase for certain topics while healthcare provider knowledge waned between the two time periods. Knowledge of Zika infection symptomatic treatment improved for the community respondents with 36% in 2017 being aware that Tylenol and rest was the primary modality of treatment compared to 22% in 2016 (*p* = 0.009). However, this value trended differently for healthcare providers: 72% in 2016 had knowledge about treatment options compared to 56% in 2017 (*p* = 0.18). More community members were able to correctly answer that a Zika vaccine was not commercially available when comparing 2017 to 2016 (87% versus 76%, *p* = 0.048); however the opposite trend was seen among healthcare providers (84% in 2017 versus 98% in 2016, *p* = 0.048). When questioned about the common breeding site of the* Aedes* mosquito, 93% of community members were able to correctly identify that the mosquito breeds near standing water compared to 80% in 2016 (*p* = 0.007); there was a small trend in the opposite direction for healthcare provider respondents (90% in 2017 versus 96% in 2016, *p* = 0.38).

Attitudes towards Zika infection changed over time (Table 3). The overall concern among community members for Zika infection being a serious problem in the respondents' home area decreased in 2017 survey administration (69% in 2017 versus 87% in 2016, *p* ≤ 0.0001). This trend was similar for healthcare providers (80% versus 84%, *p* = 0.18). When asked if Zika virus prevalence makes them worried for their families, less community members (29% versus 35%, *p* = 0.02) and healthcare providers (24% versus 30%, *p* = 0.18) were concerned in 2017. Additionally, more community members believed that a vaccine against Zika virus infection was important when comparing 2017 to 2016 (89% versus 80%, *p* = 0.095); notably, this trend was not witnessed among healthcare providers (87% versus 89%, *p* = 0.03). With regard to screening all pregnant patients for Zika exposure or infection, 61% of community members in both rounds of surveys believed that this should be a routine prenatal policy (*p* = 0.0083). Notably, more healthcare providers believed that all pregnant mothers should be screened when comparing 2017 to 2016 (53% versus 46%, *p* = 0.085). Overall concern among community members for Zika virus transmission through sexual activity remained unchanged; however a decline in concern was noted among healthcare providers (70% in 2017 versus 82% 2016, *p* = 0.089). Approximately 36% of community members were not concerned about having a child with microcephaly secondary to a Zika infection, which was an improvement from 2016 (26%, *p* = 0.01); a similar trend was noted for healthcare provider respondents over this time period (33% in 2017 versus 46% in 2016, *p* = 0.02). When asked about having a child with learning disabilities due to a Zika infection, less community members (26% in 2017 versus 37% in 2016, *p* = 0.009) and healthcare providers (34% in 2017 versus 40% in 2016, *p* = 0.04) were concerned by the second round of survey distribution.

Practices related to Zika changed over time as well (Table 4). The overall trend in the second cohort compared to the first was that less community members planned to restrict travel to Zika-endemic areas (37% versus 49%, *p* = 0.048). A similar trend was noted among healthcare professionals, although not statistically significant (35% in 2017 versus 47% in 2016, *p* = 0.19). When asked if respondents would take protective measures against mosquito infections if traveling to a Zika-endemic area, there was no statistically significant change in planned practice from 2016 to 2017 for both community members and healthcare providers. Furthermore, the overall trend was noted by 2017 that both healthcare providers and community members alike would plan for greater mosquito preventive measures within the US, including greater use of insect repellent and sleeping under a mosquito net; there was no change in anticipated practice of using screens on windows or doors. When community members were asked if they were to go to an area where Zika virus is spread and were pregnant, less would refrain from intercourse during the pregnancy (5% in 2017 versus 10% in 2016, *p* = 0.13); a similar trend was seen if their partners traveled to a Zika-endemic area (8% versus 14%, *p* = 0.13). When healthcare providers responded to these same questions, there was no identifiable trend or statistically significant association. Although statistically insignificant, less community members plan to use condoms during the entire pregnancy if they were to go to an area where Zika virus is spread and are pregnant (11% in 2017 versus 4% in 2016, *p* = 0.26); a similar trend was noted if their partners went to a Zika-endemic area (8% versus 3%, *p* = 0.31). Again, when healthcare providers responded to these questions, there was no identifiable trend or statistically significant association.

When comparing physician healthcare providers with nonphysician healthcare providers, notable differences occurred between the first and second rounds of survey administration. More physicians than nonphysicians were able to correctly identify symptoms of Zika infection in the first round of surveys (79% versus 65%, resp., *p* = 0.002) as well as correctly identify symptomatic treatment of Zika infection (85% versus 52%, *p* = 0.001). These knowledge gaps closed during the second round of surveys, with approximately the same amount of both physicians and nonphysician providers able to identify symptoms (76% and 80%, *p* = 0.02) and correct treatment (61% and 54%, *p* = 0.007). However, as can be noted from these data, more physicians were aware of the correct treatment for the virus in the first round (85%) than in the second round (61%). Interestingly, in the first round, fewer physicians than nonphysicians were aware that Zika virus can be transmitted sexually from a man to a woman (88% versus 96%, *p* = 0.001), which remained true in the second round (79% versus 86%, *p* = 0.25), although not statistically significant. Fewer physicians than nonphysicians were worried for their families due to Zika virus during the first round of surveys as well (21% versus 43%, *p* = 0.02), while the opposite was true in the second round (27% of physicians versus 21% of nonphysician providers, *p* = 0.26). Similarly, fewer physicians than nonphysicians were worried about having a child with microcephaly related to Zika infection (41% versus 65%, *p* = 0.0005) or having a child with disabilities related to Zika infection (41% versus 70%, *p* = 0.001) in the first round. In the second round, however, these groups responded much more comparably; both physician and nonphysician providers alike were similarly worried about having a child with microcephaly (61% versus 59%, *p* = 0.30) or having a child with disabilities related to Zika infection (55% versus 59%, *p* = 0.44). As a general trend, the answers to most of the attitude and practice questions related to Zika virus and protection against it indicated that physicians were less concerned overall about the virus compared to nonphysician healthcare providers in both rounds of surveys, with the difference being less noticeable in the second round, but the majority of these were not statistically significant.

## 4. Discussion

When comparing the community member results from the 2017 round of surveys to those of 2016, it is clear that there was an improvement over time in baseline knowledge with regard to Zika virus and infection. These respondents were more aware of Zika virus existence, virus vector, infectious symptoms, virus incubation period, and treatment options. Additionally, community members were more knowledgeable with regard to commercial vaccine availability, modes of transmission, mosquito breeding sites and prevention measures, and status of locally acquired Zika infections. More of these respondents were also aware of possible pregnancy and neonatal neurologic comorbidities resulting from a Zika infection. Weaker knowledge areas during both rounds of survey administration included the safety of mosquito prevention medications during pregnancy, including DEET and permethrin. DEET, the topical insect repellent, has been endorsed by the US Environmental Protection Agency to have low acute toxicity without appearing to pose a significant health concern to humans when used as directed [10]. Although some limited epidemiologic data suggests that DEET use in pregnancy may adversely affect learning and behavior, the evidence for this remains low [11]. Accordingly, permethrin is largely considered safe in pregnancy. While there are no randomized-controlled studies that distinguish outcomes based on human exposure during pregnancy, most retrospective studies find no adverse perinatal outcomes after permethrin use [12]. These results reveal that information regarding medication safety during pregnancy is not being translated to the general public appropriately, as most patients answered incorrectly or “I do not know” to these questions. Thus, increased counseling for healthcare providers is required on behalf of their patients. The overall improvement in knowledge among the community members that participated in our survey is promising, as this may parallel the general trend in Zika virus knowledge of general population as a whole. Since the majority of our survey respondents prefer to obtain their medical information from the Internet and healthcare providers (Table 1), this supports the notion that these resources have served as effective avenues for disseminating medical knowledge over the past year.

While there were notable improvements in healthcare provider knowledge during the survey periods, there were also areas where knowledge remained stagnant, or even decreased. More healthcare providers could properly identify Zika virus symptoms in 2017 compared to 2016, and there is high baseline knowledge among healthcare providers regarding mosquito prevention strategies as well as the links between virus infection and microcephaly. However, while the percentage of healthcare providers that were aware of locally acquired Zika infections within the US increased between survey rounds, this percentage was still less than 90% even in 2017. While the first documented cases of Zika infection within the US were from June to August 2016 [13] and the end of data collection for the first round of surveys was August 3, 2016, the second round of surveys occurred more than six months after the first local Zika virus infection was reported. Thus, increased healthcare provider awareness of the status of Zika virus infections within the US is warranted, as this percentage should ideally near 100%. Additionally, only about one-half of healthcare providers were able to report the percentage of infected individuals that will display symptoms. This gap in provider knowledge may lead to inadvertent patient harm including improper Zika screening, as well as inadequate patient counseling. Moreover, our survey discovered alarming areas with declining healthcare provider knowledge. Specifically, less healthcare providers were aware of DEET and permethrin safety in pregnancy in 2017 when compared to 2016, in parallel to a greater overall uncertainty with regard to medication safety as demonstrated by more “I do not know” responses. One reason for this may be that, as the initial media exposure about Zika virus subsided, new and novel information about the virus and its spread was less ubiquitous and therefore fewer providers were well informed during the 2017 survey administration. This decline in knowledge may have detrimental impact on patient counseling about safe virus prevention practices. Accordingly, there were similar declines in healthcare provider knowledge regarding options available for symptomatic treatment of Zika infections, awareness of a commercially available vaccine, and transmission of Zika virus vertically (from mother to fetus) and through sexual intercourse.

The results of the “attitude” questions between both survey periods reveal a tendency among both community members and healthcare providers to feel less concerned about Zika infection than they had previously. According to our results, there is decreased concern among both groups of survey respondents regarding concern for family members, risk of local infection, and transmission to unborn fetuses as well as postpartum neurologic outcomes for newborns. Interestingly, while the percentage of healthcare providers that were aware that Zika virus could be transferred through sexual intercourse or from mother to fetus decreased between surveys, there was a parallel decrease in concern for these outcomes among healthcare providers. The general trend of the public being less concerned about Zika virus likely derives from several ideas. First, the WHO lifted the declaration of Zika as a public health emergency of international concern, which may have contributed to alleviating fears among the general public [14]. Also, as more information is learned and distributed about Zika virus to healthcare professionals and the community alike, this likely helps to relieve anxiety and fear among the public. However, there are areas in our survey that limit our ability to fully extrapolate “attitude” information. For instance, the question “Are you concerned about having a child with learning disabilities due to a Zika infection?” does not clarify the timing of infection in relation to the pregnancy (i.e., infection before, during, or after pregnancy). Additionally, there was an upward shift among community members and healthcare providers alike in terms of belief in the need for universal Zika infection screening for pregnant women. However, we did not specify how we should screen (i.e., via questionnaire, by symptom or recent travel, or by serum testing) or when we should screen (prepregnancy versus trimester-specific screening); thus, respondents may have interpreted this question differently.

There are interesting trends to be noted when analyzing the results of the “practice” questions between both survey periods. The 2017 round of survey responses alludes to an overall decreased concern among community members with regard to planned travel to Zika-endemic areas, whether within the US or to an international site. However, it is important to review these results within the context of the demographics of the surveyed population. A majority (greater than 50%) of the survey respondents were not pregnant, nor were they planning on getting pregnant. Accordingly, there may have been an increased hesitancy for travel if a greater percentage of respondents were in a pregnancy-planning stage of their lives. Additionally, there appears to be a shift towards increased practice of mosquito prevention within the United States among community members and healthcare providers alike. Interestingly, this was not replicated when asked about mosquito prevention practices if planning to travel to Zika-endemic areas. As more confirmed cases of Zika infection occur within the United States, this may encourage the general public to begin to practice routine mosquito prevention practices, rather than only associated with travel.

There are other limitations of our study that have been identified. One limitation is that the answers to some of the questions in the survey may have changed throughout survey administration. For example, one question asked the respondent whether any documented cases of Zika virus transmission in the US have occurred. In the first survey administration, the answer to this question changed as initially there were no documented cases, but towards the end of the survey administration period there had been several cases noted. This changes the distribution of answers and the ability to compare this to the second group. Additionally, when asked about vaccine availability, there may be potential confusion among healthcare provider respondents with regard to the current status of an “approved” Zika virus vaccine. At the time of survey administration, investigational vaccines were undergoing clinical trial [15] and this ambiguity may have contributed to the distribution of answers that were obtained for this question. Another limitation of our study is that the total number of respondents in the first group is significantly less than in the second group, so the ability to detect statistically significant differences within this group when comparing the first group to the second group is diminished. Accordingly, the cohort composition was different between the two time periods, which would limit our ability to measure change longitudinally. It is also unknown exactly how many potential participants the survey was sent to via email and how many were approached in clinic, as the number of participants on each listserv changes frequently and the number of participants approached in the clinic was not recorded. This discrepancy in the number of participants as well as potential differences in baseline characteristics of responders versus nonresponders to the survey may contribute an element of participation bias. However, it can be estimated that the number of potential participants was about 800 by analyzing current listserv cohorts and from an estimation of those approached in clinic. Another limitation is that the sampling of respondents was convenience sampling, meaning that the survey was distributed both in the clinic and via the online link based on the potential participants' ease of approachability and willingness to respond to the survey. It is important to note that our survey also did not stratify healthcare providers by their specific profession (i.e., ultrasound technician versus nurse versus other healthcare professionals) and only asked about physician versus nonphysician status; this may have played a role in skewing the survey results, as the second group of healthcare providers may not represent the same expertise or experience as the first group of surveyed providers.

## 5. Conclusion

Zika virus is a new consideration in the field of congenitally acquired diseases. This study aimed to address the evolving knowledge, attitudes, and practice regarding Zika virus during a tumultuous time of many unknowns for patients and providers alike. From this study, we can extrapolate that, as more information is gathered about the virus, more questions will be answered and there will be fewer fears regarding the virus. This data can be used to identify gaps in knowledge at this time about Zika virus and prepare medical providers to offer counseling regarding safety and preventive practices.

## Figures and Tables

**Table 1:** Demographic characteristics of survey respondents.

Characteristic	Details	2016 Survey Frequency (%)	2017 Survey Frequency (%)
Gender	Male	28/174 (16)	40/277 (14)
	Female	129/174 (74)	215/277 (78)
	No answer	17/174 (10)	22/277 (8)

Marital status	Single	52/174 (30)	125/277 (45)
	Partnered	18/174 (10)	33/277 (12)
	Married	79/174 (45)	89/277 (32)
	Other	25/174 (15)	30/277 (11)

Highest level of education	High School	12/174 (7)	0/277 (0)
	College	32/174 (18)	84/277 (30)
	Graduate school	60/174 (34)	112/277 (40)
	Post-Grad	53/174 (30)	60/277 (22)
	No answer	17/174 (10)	21/277 (8)

Average income	<$25,000	36/174 (21)	90/277 (32)
	$25,000–50,000	12/174 (7)	30/277 (11)
	$50,000–75,000	31/174 (18)	44/277 (16)
	$75,000–100,000	17/174 (10)	24/277 (9)
	>$100,000	39/174 (22)	39/277 (14)
	Prefer not to answer	17/174 (10)	29/277 (10)
	No answer	22/174 (13)	21/277 (8)

Ethnicity	Caucasian	92/174 (53)	188/277 (68)
	African American	27/174 (15)	14/277 (5)
	Hispanic	11/174 (6)	11/277 (4)
	Asian	18/174 (10)	31/277 (11)
	Other	9/174 (5)	12/277 (4)
	No answer	17/174 (10)	21/277 (8)

County of birth	US	136/174 (78)	219/277 (79)
	Other	21/174 (12)	36/277 (13)
	No answer	17/174 (10)	22/277 (8)

Country of residency	US	156/174 (90)	252/277 (91)
	Other	0/174 (0)	2/277 (1)
	No answer	18/174 (10)	23/277 (8)

State of residence	Washington, DC	67/174 (39)	73/277 (26)
	Maryland	30/174 (17)	32/277 (12)
	Virginia	29/174 (17)	52/277 (19)
	Other state	10/174 (6)	60/277 (22)
	No answer	38/174 (22)	60/277 (22)

Type of insurance	Medicare	8/174 (5)	2/277 (1)
	Medicaid	11/174 (6)	4/277 (1)
	Private	134/174 (77)	244/277 (88)
	None	1/174 (1)	4/277 (1)
	No answer	20/174 (11)	23/277 (8)

Healthcare provider status	Primary Care physician	10/174 (6)	8/277 (3)
	OB/GYN physician	16/174 (9)	15/277 (5)
	Other physician	8/174 (5)	10/277 (4)
	Non-physician provider	23/174 (13)	56/277 (20)
	Non-provider	117/174 (67)	188/277 (68)

Pregnancy status	Not pregnant	93/174 (53)	205/277 (74)
	Pregnant	32/174 (18)	10/277 (4)
	Planning to be pregnant	8/174 (5)	9/277 (3)
	Postpartum (<6 wk)	3/174 (2)	1/277 (0)
	Not applicable	21/174 (12)	31/277 (11)
	No answer	17/174 (10)	21/277 (8)

Primary source of healthcare information	Health publication	30/174 (17)	37/277 (13)
	Internet	55/174 (32)	100/277 (36)
	Social Media	2/174 (1)	4/277 (1)
	Healthcare provider	36/174 (21)	56/277 (20)
	Newspaper	6/174 (3)	12/277 (4)
	At work	7/174 (4)	21/277 (8)
	Other	20/174 (11)	26/277 (9)
	No answer	18/174 (10)	21/277 (8)

Where would you like to obtain your healthcare information	Text Message	7/174 (4)	8/277 (3)
	Health publication	31/174 (18)	43/277 (16)
	Internet	45/174 (26)	90/277 (32)
	Social Media	4/174 (2)	15/277 (5)
	Healthcare provider	43/174 (25)	61/277 (22)
	Newspaper	8/174 (5)	9/277 (3)
	Other	19/174 (11)	30/277 (11)
	No answer	17/174 (10)	21/277 (8)

**Table 2:** Responses to “knowledge” questions regarding Zika virus disease and infection.

	Community Member Responses			Healthcare Provider Responses		
	2016 Survey (%)	2017 Survey (%)	95% CI	2016 Survey (%)	2017 Survey (%)	95% CI
*Are you aware of Zika virus?*						
Yes	91/97 (94)	165/166 (99)	0.02	57/57 (100)	87/89 (98)	0.25
No	2/97 (2)	0/166 (0)		0/57 (0)	2/89 (2)	
No answer	4/97 (4)	1/166 (1)		0/57 (0)	0/89 (0)	
*How is the Zika virus spread?*						
Fly bite	0/97 (0)	1/166 (1)		0/57 (0)	0/89 (0)	
Mosquito Bite	92/97 (95)	163/166 (98)	0.19	56/57 (98)	87/89 (98)	0.24
Do not know	1/97 (1)	1/166 (1)		1/57 (2)	0/89 (0)	
No answer	4/97 (4)	1/166 (1)		0/57 (0)	2/89 (2)	
*Which of the following is NOT a common symptom of Zika virus infection?*						
Fever	2/97 (2)	1/166 (1)		0/57 (0)	2/89 (2)	
Rash	3/97 (3)	5/166 (3)		4/57 (7)	2/89 (2)	
Pneumonia	45/97 (46)	107/166 (64)	0.019	42/57 (74)	70/89 (79)	0.19
Joint Pain	2/97 (2)	2/166 (1)		1/57 (2)	0/89 (0)	
Conjunctivitis (red eyes)	15/97 (15)	28/166 (17)		5/57 (9)	3/89 (3)	
Do not know	26/97 (27)	22/166 (13)		5/57 (9)	12/89 (13)	
No answer	4/97 (4)	1/166 (1)		0/57 (0)	2/89 (2)	
*What percentage of people infected with Zika virus will have symptoms?*						
20%	38/97 (39)	76/166 (46)		29/57 (51)	47/89 (53)	
40%	16/97 (16)	28/166 (17)	0.24	11/57 (19)	11/89 (12)	0.52
60%	3/97 (3)	7/166 (4)		2/57 (4)	1/89 (1)	
80%	1/97 (1)	1/166 (1)		0/57 (0)	1/89 (1)	
Do not know	34/97 (35)	53/166 (32)		15/57 (26)	27/89 (30)	
No answer	5/97 (5)	1/166 (1)		0/57 (0)	2/89 (2)	
*What is the treatment for Zika virus infection?*						
Antibiotics	5/97 (5)	10/166 (6)		0/57 (0)	2/89 (2)	
Antivirals	26/97 (27)	53/166 (32)		0/57 (0)	3/89 (3)	
Antifungals	0/97 (0)	0/166 (0)		7/57 (12)	19/89 (21)	
Tylenol and rest	21/97 (22)	59/166 (36)	0.009	41/57 (72)	50/89 (56)	0.18
Do not know	41/97 (42)	43/166 (26)		9/57 (16)	15/89 (17)	
No answer	4/97 (4)	1/166 (1)		0/57 (0)	2/89 (2)	
*Has anyone been infected with Zika virus in the US?*						
Yes	63/97 (65)	151/166 (91)	0.0001	35/57 (61)	75/89 (84)	0.002
No	20/97 (21)	7/166 (4)		18/57 (32)	7/89 (8)	
Do not know	9/97 (9)	7/166 (4)		4/57 (7)	5/89 (5)	
No answer	5/97 (5)	1/166 (1)		0/57 (0)	2/89 (2)	
*Has anyone been infected with Zika virus by traveling from the US to known Zika areas outside the US?*						
Yes	84/97 (87)	151/166 (91)	0.24	53/57 (93)	83/89 (93)	0.64
No	4/97 (4)	6/166 (3)		1/57 (2)	1/89 (1)	
Do not know	5/97 (5)	8/166 (5)		3/57 (5)	3/89 (3)	
No answer	4/97 (4)	1/166 (1)		0/57 (0)	2/89 (2)	
*Is there an approved vaccine for Zika virus?*						
Yes	2/97 (2)	6/166 (4)		1/57 (2)	4/89 (4)	
No	74/97 (76)	144/166 (87)	0.048	56/57 (98)	75/89 (84)	0.048
Do not know	17/97 (17)	14/166 (8)		0/57 (0)	8/89 (9)	
No answer	4/97 (4)	2/166 (1)		0/57 (0)	2/89 (2)	
*Is there a link between Zika virus and the birth defect microcephaly (small head)?*						
Yes	87/97 (90)	157/166 (95)	0.09	57/57 (100)	87/89 (98)	0.25
No	0/97 (0)	1/166 (1)		0/57 (0)	0/89 (2)	
Do not know	5/97 (5)	7/166 (4)		0/57 (0)	0/89 (0)	
No answer	5/97 (5)	1/166 (1)		0/57 (0)	2/89 (2)	
*Can an infected man transfer Zika virus to a woman through sexual activity?*						
Yes	74/97 (76)	144/166 (87)	0.12	52/57 (91)	74/89 (83)	0.4
No	1/97 (1)	4/166 (2)		3/57 (5)	6/89 (7)	
Do not know	14/97 (14)	13/166 (8)		1/57 (2)	3/89 (3)	
It is unknown	3/97 (3)	2/166 (1)		1/57 (2)	1/89 (1)	
No answer	5/97 (5)	3/166 (2)		0/57 (0)	5/89 (5)	
*Can an infected mother transfer Zika virus to her unborn baby?*						
Yes	67/97 (69)	144/166 (87)	0.008	52/57 (91)	75/89 (84)	0.34
No	3/97 (3)	2/166 (1)		1/57 (2)	1/89 (1)	
Do not know	19/97 (19)	12/166 (7)		3/57 (5)	4/89 (5)	
It is unknown	3/97 (3)	5/166 (3)		1/57 (1)	3/89 (3)	
No answer	5/97 (5)	3/166 (2)		0/57 (0)	6/89 (6)	
*What is the common breeding site for the mosquito that spreads Zika virus?*						
standing water	78/97 (80)	155/166 (93)	0.007	55/57 (96)	80/89 (90)	0.38
running water	0/97 (0)	1/166 (1)		0/57 (0)	1/89 (1)	
salt water	0/97 (0)	1/166 (1)		0/57 (0)	0/89 (0)	
Do not know	13/97 (13)	6/166 (3)		2/57 (4)	5/89 (6)	
No answer	6/97 (6)	3/166 (2)		0/57 (0)	3/89 (3)	
*Is DEET safe in pregnancy?*						
Yes	24/97 (25)	34/166 (20)	0.19	28/57 (49)	30/89 (34)	0.05
No	29/97 (30)	60/166 (36)		19/57 (33)	26/89 (29)	
Do not know	38/97 (39)	69/166 (42)		10/57 (18)	29/89 (33)	
No answer	6/97 (6)	3/166 (2)		0/57 (0)	4/89 (4)	
*Is permethrin safe in pregnancy?*						
Yes	10/97 (10)	19/166 (11)	0.18	19/57 (33)	19/89 (21)	0.17
No	10/97 (10)	20/166 (12)		9/57 (16)	19/89 (21)	
Do not know	69/97 (71)	123/166 (74)		29/57 (51)	47/89 (53)	
No answer	8/97 (8)	4/166 (2)		0/57 (0)	4/89 (4)	
*Where should you apply insect repellent?*						
Under sunscreen	11/97 (11)	24/166 (14)		7/57 (12)	16/89 (18)	
Over sunscreen	43/97 (44)	87/166 (52)	0.05	38/57 (67)	38/89 (43)	0.04
Do not know	35/97 (36)	52/166 (31)		11/57 (19)	32/89 (36)	
No answer	8/97 (8)	3/166 (2)		1/57 (1)	3/89 (3)	
*If infected with Zika virus, when is one most likely to infect others?*						
First week of symptoms	20/97 (21)	51/166 (31)	0.09	26/57 (46)	29/89 (33)	0.24
Second week of symptoms	8/97 (8)	13/166 (8)		4/57 (7)	6/89 (6)	
Third week of symptoms	2/97 (2)	6/166 (4)		2/57 (4)	1/89 (1)	
Do not know	60/97 (62)	93/166 (56)		25/57 (44)	50/89 (56)	
No answer	7/97 (7)	3/166 (2)		1/57 (0)	3/89 (3)	
*These precautions will help to reduce the risk of mosquito bites (yes/no)*						
Remove standing water	80/97 (82)	154/166 (93)	0.015	56/57 (98)	81/89 (91)	0.29
Sleep under a mosquito net	76/97 (78)	160/166 (96)	0.0001	54/57 (95)	83/89 (93)	0.15
Use screens on windows and doors	82/97 (85)	160/166 (96)	0.0049	56/57 (98)	84/89 (94)	0.19

**Table 3:** Responses to “attitude” questions regarding Zika virus disease and infection.

	Community Member Responses			Healthcare Provider Responses		
	2016 Survey (%)	2017 Survey (%)	95% CI	2016 Survey (%)	2017 Survey (%)	95% CI
*Do you think Zika virus is a serious problem in your area?*						
Yes	13/97 (13)	17/166 (10)		8/57 (14)	10/89 (11)	
No	67/97 (69)	144/166 (87)	0.0001	48/57 (84)	71/89 (80)	0.18
Do not know	9/97 (9)	0/166 (0)		1/57 (2)	1/89 (1)	
No answer	8/97 (8)	5/166 (3)		0/57 (0)	7/89 (7)	
*Does the Zika virus make you worried for your family?*						
Yes	34/97 (35)	48/166 (29)	0.02	17/57 (30)	21/89 (24)	0.18
No	50/97 (52)	109/166 (66)		36/57 (63)	61/89 (69)	
Do not know	5/97 (5)	1/166 (1)		2/57 (4)	0/89 (0)	
No answer	8/97 (8)	8/166 (5)		2/57 (4)	7/89 (7)	
*Do you think that a vaccine against Zika virus is important?*						
Yes	78/97 (80)	148/166 (89)	0.095	51/57 (89)	77/89 (87)	0.03
No	4/97 (4)	8/166 (5)		3/57 (5)	0/89 (0)	
Do not know	7/97 (7)	5/166 (3)		3/57 (5)	5/89 (6)	
No answer	8/97 (8)	5/166 (3)		0/57 (0)	7/89 (8)	
*Should all newly pregnant mothers be screened for Zika?*						
Yes	59/97 (61)	101/166 (61)	0.0083	26/57 (46)	47/89 (53)	0.085
No	28/97 (19)	52/166 (31)		27/57 (47)	30/89 (34)	
Do not know	12/97 (12)	8/166 (5)		4/57 (7)	5/89 (6)	
No answer	8/97 (8)	5/166 (3)		0/57 (0)	7/89 (8)	
*Are you concerned about Zika virus spreading through sexual activity?*						
Yes	65/97 (67)	120/166 (72)	0.18	47/57 (82)	62/89 (70)	0.089
No	17/97 (18)	34/166 (20)		9/57 (16)	15/89 (17)	
Do not know	7/97 (7)	7/166 (4)		1/57 (2)	5/89 (6)	
No answer	8/97 (8)	5/166 (3)		0/57 (0)	7/89 (8)	
*Are you concerned about Zika spreading from mother to unborn baby?*						
Yes	70/97 (72)	123/166 (74)		53/57 (93)	76/89 (86)	
No	9/97 (9)	30/166 (18)	0.018	2/57 (4)	4/89 (4)	0.17
Do not know	8/97 (8)	8/166 (5)		2/57 (4)	2/89 (2)	
No answer	10/97 (10)	5/166 (3)		0/57 (0)	7/89 (8)	
*Are you concerned about having a child with microcephaly (small head) related to Zika infection?*						
Yes	56/97 (58)	98/166 (59)		29/57 (51)	53/89 (60)	
No	25/97 (26)	60/166 (36)	0.01	26/57 (46)	29/89 (33)	0.02
Do not know	7/97 (7)	3/166 (2)		2/57 (4)	0/89 (0)	
No answer	9/97 (9)	5/166 (3)		0/57 (0)	7/89 (8)	
*Are you concerned about having a child with learning disabilities related to Zika infection?*						
Yes	54/97 (56)	95/166 (57)		30/57 (53)	51/89 (57)	
No	25/97 (26)	61/166 (37)	0.009	23/57 (40)	30/89 (34)	0.04
Do not know	9/97 (9)	4/166 (2)		3/57 (5)	0/89 (0)	
No answer	9/97 (9)	6/166 (4)		1/57 (2)	8/89 (9)	
*If you went to an area where Zika is spread and are a man, are you concerned about spreading Zika sexually to your partner?*						
Yes	10/97 (10)	21/166 (13)		13/57 (23)	21/89 (24)	
No	7/97 (7)	9/166 (5)	0.37	0/57 (0)	2/89 (2)	0.6
Do not know	3/97 (3)	5/166 (3)		2/57 (4)	2/89 (2)	
Not applicable	68/97 (70)	125/166 (75)		40/57 (70)	57/89 (64)	
No answer	9/97 (9)	6/166 (4)		2/57 (3)	7/89 (8)	

**Table 4:** Responses to “practice” questions regarding Zika virus disease and infection.

	Community Member Responses			Healthcare Provider Responses		
	2016 Survey (%)	2017 Survey (%)	95% CI	2016 Survey (%)	2017 Survey (%)	95% CI
*Do you plan to restrict your travel to a Zika endemic area?*						
Yes	48/97 (49)	61/166 (37)	0.048	27/57 (47)	31/89 (35)	0.19
No	34/97 (35)	86/166 (52)		25/57 (44)	47/89 (53)	
Do not know	7/97 (7)	12/166 (7)		4/57 (7)	4/89 (5)	
No answer	8/97 (8)	7/166 (4)		1/57 (2)	7/89 (8)	
*If you travel to a Zika endemic area, will you take protective measures to prevent a mosquito bite?*						
Yes	85/97 (88)	147/166 (89)	0.11	53/57 (93)	79/89 (89)	0.09
No	0/97 (0)	7/166 (4)		3/57 (5)	3/89 (3)	
Do not know	4/97 (4)	3/166 (2)		1/57 (2)	0/89 (0)	
No answer	8/97 (8)	9/166 (5)		0/57 (0)	7/89 (8)	
*Do you plan on taking protective measures against mosquito bites while in the US to prevent Zika infection?*						
Yes	52/97 (54)	96/166 (58)	0.29	24/57 (42)	36/89 (41)	0.18
No	30/97 (31)	57/166 (34)		31/57 (54)	44/89 (50)	
Do not know	7/97 (7)	6/166 (4)		2/57 (4)	2/89 (2)	
No answer	8/97 (8)	7/166 (4)		0/57 (0)	7/89 (8)	
*If you are in an area with mosquitos, you take the following preventive measures*						
Use insect repellent	73/97 (75)	138/166 (83)	0.18	47/57 (83)	77/89 (87)	0.018
Remove standing water	62/97 (64)	115/166 (69)	0.56	43/57 (75)	61/69 (69)	0.15
Sleep under a mosquito net	29/97 (30)	60/166 (36)	0.12	11/57 (19)	31/89 (35)	0.003
Use screens on windows and doors	84/97 (87)	145/166 (87)	0.16	50/57 (88)	78/89 (88)	0.01
*If you went to an area where Zika virus is spread and are pregnant, do you plan to not have sex during the entire pregnancy?*						
Yes	10/97 (10)	9/166 (5)	0.13	5/57 (9)	7/89 (8)	0.24
No	8/97 (8)	30/166 (18)		10/57 (18)	12/89 (13)	
Do not know	19/97 (20)	24/166 (14)		7/57 (12)	12/89 (13)	
Not applicable	52/97 (54)	90/166 (54)		34/57 (60)	47/89 (53)	
No answer	8/97 (8)	13/166 (8)		1/57 (2)	11/89 (12)	
*If you went to an area where Zika virus is spread and are pregnant, do you plan to use condoms during the entire pregnancy?*						
Yes	18/97 (19)	29/166 (17)	0.26	13/57 (23)	14/89 (16)	0.2
No	4/97 (4)	18/166 (11)		4/57 (7)	6/89 (7)	
Do not know	16/97 (16)	17/166 (10)		4/57 (7)	10/89 (11)	
Not applicable	51/97 (53)	89/166 (54)		35/57 (61)	49/89 (55)	
No answer	8/97 (8)	13/166 (8)		1/57 (2)	10/89 (11)	
*If your partner went to an area where Zika virus is spread and you are pregnant, do you plan to not have sex during the entire pregnancy?*						
Yes	14/97 (14)	13/166 (8)	0.13	7/57 (12)	7/89 (8)	0.067
No	6/97 (6)	25/166 (15)		11/57 (19)	10/89 (11)	
Do not know	17/97 (18)	24/166 (14)		4/57 (7)	14/89 (16)	
Not applicable	52/97 (54)	89/166 (54)		34/57 (60)	48/89 (54)	
No answer	8/97 (8)	15/166 (9)		1/57 (2)	10/89 (11)	
*If your partner went to an area where Zika virus is spread and you are pregnant, do you plan to use condoms during the entire pregnancy?*						
Yes	19/97 (20)	30/166 (18)	0.31	14/57 (25)	16/89 (18)	0.26
No	3/97 (3)	14/166 (8)		4/57 (7)	5/89 (6)	
Do not know	16/97 (16)	17/166 (10)		4/57 (7)	8/89 (9)	
Not applicable	51/97 (53)	90/166 (54)		34/57 (60)	50/89 (56)	
No answer	8/97 (8)	15/166 (9)		1/57 (2)	10/89 (11)	
*If you are thinking of becoming pregnant, do you plan to delay your pregnancy due to the Zika outbreak?*						
Yes	11/97 (11)	20/166 (12)	0.78	10/57 (18)	17/89 (19)	0.23
No	12/97 (12)	30/166 (18)		8/57 (14)	9/89 (10)	
Do not know	11/97 (11)	16/166 (10)		4/57 (7)	5/89 (6)	
Not applicable	53/97 (55)	86/166 (52)		34/57 (60)	47/89 (53)	
No answer	10/97 (10)	14/166 (8)		1/57 (2)	11/89 (12)	
